# The loss of both pUL16 and pUL21 in HSV-1-infected cells alters capsid–tegument composition, nuclear membrane architecture, cytoplasmic maturation and cell-to-cell spread

**DOI:** 10.1099/jgv.0.002083

**Published:** 2025-03-13

**Authors:** Kellen Roddy, Peter Grzesik, Barbara J. Smith, Nathan Ko, Sanjay Vashee, Prashant J. Desai

**Affiliations:** 1 Department of Oncology, Johns Hopkins University School of Medicine, Baltimore, MD, USA; 2 Department of Cell Biology, Johns Hopkins University School of Medicine, Baltimore, MD, USA; 3 Synthetic Biology and Bioenergy, J. Craig Venter Institute, Rockville, MD, USA

**Keywords:** cell-to-cell spread, cytoplasmic envelopment, herpes simplex virus, nuclear membrane architecture, pUL16, pUL21, tegument proteins

## Abstract

Previously, we had developed synthetic genomics methods to assemble an infectious clone of herpes simplex virus type-1 (HSV-1) strain KOS. To do this, the genome was assembled from 11 separate cloned fragments in yeast using transformation-associated recombination. Using this method, we generated null mutations in five tegument protein-coding genes as well as different combinations of these mutants. The single-locus mutants were all able to plaque on Vero cells. However, one multi-locus combination, ∆UL16/UL21, proved lethal for virus replication in non-permissive cells. The proteins encoded by the genes UL16 and UL21 are of interest because they are known to physically interact and are constituents of the tegument structure. Furthermore, their roles in HSV-1-infected cells are unclear. Both are dispensable for HSV-1 replication; however, in HSV-2, their mutation results in nuclear retention of assembled capsids and has activities that impact nuclear membrane integrity as well as activities of proteins that function in nuclear egress. We thus characterized these HSV-1 viruses that carry the single and double mutants. What we found was that the single mutants could replicate within cells and spread from infected to uninfected cells, albeit at significantly reduced levels. However, the double mutant (∆16/21) could not produce infectious progeny in a 24 h growth cycle and could not spread from cell to cell. Confocal microscopy of VP16-Venus expressed by these viruses as well as immunofluorescence assays for glycoprotein B showed perturbation of the nuclear membrane, which was pronounced in ∆21 and ∆16/21 infected cells. All the mutants assembled DNA-filled capsids as judged by ultrastructural analyses and sedimentation studies. Electron microscopy revealed the presence of numerous mature viruses in WT-infected cells but fewer such particles in the ∆16- and ∆21-infected cells. What we discovered is that in cells where both pUL16 and pUL21 are absent, cytoplasmic capsids were evident, but mature enveloped particles were not detected. The capsid particles isolated from all the single- and multi-locus mutant-infected cells showed significantly lower levels of incorporation of both VP16 and pUL37 when compared to the WT capsids. This reduced incorporation may be related to the loss of the integrity of the architecture of the nuclear membrane. Interestingly, the incorporation of pUL16 was not affected by the absence of pUL21 and vice versa, as judged by immunoblots. These data now show that of the tegument proteins, like the essential pUL36, pUL37 and VP16, the complex of pUL16 and pUL21 should be considered as important mediators of maturation and cell-to-cell spread of the particle.

## Introduction

The herpes simplex virus type-1 (HSV-1) virion is comprised of four structural components: an icosahedral capsid, which encloses the viral DNA genome; an electron-dense asymmetrically distributed material, which immediately surrounds the capsid and is termed the tegument; and an outer membrane or envelope, which encloses the tegument and capsid and in which the viral glycoproteins are embedded [1,4]. Capsid assembly and DNA packaging into icosahedral capsids are nuclear events. Subsequent nuclear exit and cytoplasmic envelopment involve the participation of a large and diverse set of proteins.

The tegument is one of the most complex and diverse structures of the virion both in terms of protein composition and the functions encoded by the constituents of this structure. The tegument is comprised of a dense protein network that maintains this structure even when devoid of the virus envelope or capsid [4,11]. The tegument proteins have been classified as belonging to either the inner or outer layer of the tegument based on their close association with either the capsid (inner) or envelope (outer) [2,12,16]. What has become increasingly evident is the importance of the tegument proteins in the maturation process of the enveloped virus. To date, three tegument protein residents in the mature virion have been shown to have a deleterious and complete lethal effect on the maturation process. These are VP16 [17,18], pUL36 (VP1/2) [19,22] and the product of the UL37 gene [20,23, 24]. Mutations in these three gene products result in the accumulation of unenveloped particles in the cytoplasm.

The studies presented here build on our recent experiments using the synthetic genomics assembly line to generate HSV-1 KOS genomes carrying single, double, triple, quadruple and quintuple mutations in different combinations of five genes encoding the tegument proteins pUL7, pUL51, pUL11, pUL16 and pUL21 [25]. This significant technical advance, to generate in parallel these mutant viruses, could only be done using this modular assembly method. These five genes, UL7, UL11, UL16, UL21 and UL51, have varied functions in the infected cell. Each single mutant virus was able to replicate in non-permissive cells, albeit poorly. However, combinatorial analysis of deletions in the five genes revealed ‘synthetic-lethality’ of some of the genetic mutations. Thus, it was discovered that any virus that carried a UL21 mutation in addition to the other gene was unable to replicate in Vero cells. Replication was restored in a complementing cell line that provided pUL21 in trans. Several studies have identified protein interactions between pUL7 and pUL51 [26,27] and between pUL11, pUL16 and pUL21 [27,36], but other than single mutations, many have not been probed using multiple/combinatorial mutagenesis except for the UL7-UL51 gene pair [26,37]. These five proteins are conserved in all three of the herpesvirus families, yet they are not essential, at least for HSV-1, in cell culture [26,38,45]. Investigations of UL16 and UL21 mutations in HSV-2 strains have revealed different phenotypes [46,47]. Replication of these mutant viruses is severely compromised, and ultrastructural analysis of infected cells discovered defects in nuclear egress. Studies have shown that HSV-2 pUL21 impacts the nuclear egress complex (NEC) (pUL31 and pUL34) as well as the kinase (pUS3) required for the activity of this complex [48,49]. The absence of pUL21 activity manifests in post-translational modification of the NEC proteins by pUS3, which translates to significant alterations to the architecture of the nuclear membrane. These alterations may retard nuclear exit and maturation of virus particles [48]. This perturbation of the nuclear envelope was also seen with HSV-1 strains that lack pUL21 [49]. There are additional activities ascribed to both HSV-2 pUL16 and pUL21, which include retention of the viral genome in the capsid (pUL21) [50], as well as affecting the docking of input viral capsids at the nuclear pore (both pUL16 and pUL21) [51].

In this study, we further investigated the combination of UL16 and UL21 mutations because these proteins have a documented history of physical interactions in the infected cell [28,30, 31]. The viruses carrying single mutations in these genes replicated in non-permissive cells, albeit poorly. The double mutant virus displayed significant impairment in virus replication; there was no virus production within cells and no virus spread from infected to uninfected cells. There were also visually observed deformations in the architecture of the nuclear membrane, especially in cells infected with ∆21 and ∆16/21 viruses. When infected cells were examined further, it was evident that the ∆16/21 virus assembled DNA-filled capsids, and these particles were also observed in the cytoplasmic compartment. Envelopment of the ∆16 and ∆21 mutants in the cytoplasm was reduced compared to WT-infected cells, whereas envelopment of the ∆16/21 particles was not observed. Further examination of the DNA-filled C-capsids revealed a significant reduction in the capsid association of VP16 (outer tegument) and pUL37 (inner-tegument) proteins. The loss of the functional pUL16–pUL21 complexes has a significant impact on virus maturation, thus revealing the complex nature of how HSV-1 capsids mature into infectious particles.

## Methods

### Cells and viruses

Vero cells, transformed Vero cell lines (G5-9) and retinal pigment epithelial cells (RPE-1) were all grown in minimal essential medium (alpha medium, Gibco Invitrogen) supplemented with 10% FBS (Gibco Invitrogen) and passaged as described previously [52]. G5-9 is a subclone of the original G5 cell line isolated by Desai *et al.* [53]. This cell line carries a genomic fragment that includes UL16 and UL21 and thus complements mutants that carry deletions in these two genes. All stocks of HSV-1 viruses were amplified as also described by Desai *et al*. [52].

### Antibodies

Antibodies to VP16 (LP1) were generated by Professor Tony Minson (University of Cambridge). This is a well-characterized monoclonal antibody to this protein and has a significant citation record. Rabbit antibodies to pUL16 and pUL21 were made by John Wills (University of Pennsylvania, Hershey), and these have strong validation in the literature. Rabbit antibody to VP23 was generated by our lab using whole protein purified from capsid preparations and has demonstrated specificity [53]. Monoclonal antibody to glycoprotein D (gD) (clone DL6) was generated by Dr. Cohen (University of Pennsylvania) and kindly provided to us by David Johnson (Oregon Health Sciences Center). This is a well-established antibody to gD. Antibody to pUL37 (rabbit polyclonal) was generated by Frank Jenkins (University of Pittsburgh). Mouse monoclonal antibody MCA406, which recognizes both VP21 and VP22a, was purchased from Serotec Inc. GFP rabbit antibody (ab183734) was purchased from Abcam. Mouse monoclonal antibody to glycoprotein B (gB) (B6 clone) was produced by Joseph Glorioso (University of Pittsburgh) [54], and rabbit antibody to gB was produced by Tom Holland (Wayne State University). Beta-actin antibody (clone 66009) was purchased from Proteintech.

### Cre excision

For the Cre excision, we used 2.5 µg of the DNA in a 50 µl volume reaction and used Cre enzyme (two units/μl) (NEB). This was incubated at 37 °C for 30 min, and then, the enzyme was heat-inactivated at 70 °C for 10 min. The whole 50 µl reaction was transfected into Vero or G5-9 cells using X-tremeGENE transfection reagent (Sigma-Aldrich) using the protocol previously [25]. The transfection was harvested 3 days post-infection and sonicated to generate an infected cell lysate. This was serially diluted and used to infect cells in 96-well trays. Single plaques isolated were amplified and checked by Phire Hot Start II polymerase (Invitrogen) PCR assays to check for excision as described previously [25,55]. Positives were amplified further to generate high-titre working stocks.

### Growth curves

Vero or G5-9 cells (5×10^5^) in 12-well trays were infected at a multiplicity of infection (MOI) of 10 p.f.u./cell. The virus was adsorbed to the cells in a minimal volume, and the virus inoculum was removed after 1 h and overlaid with growth media. The cells were harvested at 2, 6 and 24 h post-infection. Cells were frozen/thawed three times and sonicated, and the virus progeny was titred on G5-9 monolayers.

### Infectious centre plaque assay

Vero or G5-9 cells (5×10^5^) in 12-well trays were infected with 100 p.f.u. for 1 h and then overlaid with growth media. At 2, 24, 48 and 72 h after infection, the cells were washed with PBS and trypsinized. The infected cells and dilutions of these cells were then mixed with uninfected G5-9 cells and incubated (3 days) to determine the number of infectious centres in each well.

### Western blot analysis of infected cell lysates

Vero cells (5×10^5^) were infected at an MOI of 10 p.f.u./cell and harvested 24 h post-infection. Cell pellets were lysed in 2X Laemmli buffer, and 10% of this sample was resolved using NuPAGE 4–12% Bis-Tris gels (Invitrogen) and transferred to nitrocellulose membranes using the iBlot2 system (Invitrogen) as described by Luitweiler *et al*. [56]. Rabbit antibodies to HSV antigens were used at a dilution of 1 : 500. Blots were processed using the enhanced chemiluminescence kit (GE Healthcare) or Clarity chemiluminescence kit (Bio-Rad) according to the manufacturer’s protocol and imaged using the iBright 1500 Imager (Invitrogen).

### Fluorescence light microscopy imaging

For confocal imaging, RPE-1 or Vero cells (5×10^5^) were seeded in a four-well borosilicate glass bottom chamber slide (Lab-Tek). Cells were infected with each virus at an MOI of 10 p.f.u./cell and overlaid with FluoroBrite DMEM (Thermo Fisher) supplemented with 1% FBS. At 12 (RPE-1) or 20 h (Vero) after infection, cells were imaged on a Zeiss LSM 510 or LSM 800 confocal microscope using a 63X objective. Immunofluorescence assays were carried out as described by Desai *et al*. [57].

### Transmission electron microscopy (TEM)

Vero cells (5×10^5^ cells) in 12-well tissue culture trays were infected at an MOI of 10 p.f.u./cell and processed for TEM experiments [23]. Infected cells were processed 16 h post-infection. Samples were fixed in 2.5% glutaraldehyde, 3 mM MgCl_2_, in 0.1 M sodium cacodylate buffer, pH 7.2 for overnight at 4 °C. After buffer rinse, samples were postfixed in 1% osmium tetroxide in 0.1 M sodium cacodylate buffer (1 h) on ice in the dark. Following a DH_2_O rinse and en bloc staining in 0.75% uranyl acetate for 3 h, samples were dehydrated in a graded series of ethanol and embedded in Eponate resin overnight at 60 °C. Thin sections, 60–90 nm, were cut with a diamond knife on a Leica UltracutE ultramicrotome and picked up with 2×1 mm formvar-coated copper slot grids. Grids were stained with 2% uranyl acetate (aq.) and 0.4% lead citrate before imaging on a Hitachi 7600 TEM at 80 kV equipped with an AMT XR80 CCD.

### Capsid purification and analysis of composition

Vero cells (20×10^6^) in 100 mm tissue culture dishes were infected at an MOI of 5 and harvested after 24 h. Capsids from infected cells were released by treating infected cell pellets with 2X capsid lysis buffer (CLB) (1 M NaCl, 2% Triton X-100, 20 mM Tris-HCl pH 7.5, 2 mM EDTA pH 8) [53] followed by 30 s sonication. Next, capsids were separated using rate-velocity sedimentation on a 20–50% sucrose gradient. Capsid bands were visualized using light scattering, and the C-capsid band was harvested by side puncture. The capsid fractions were TCA (trichloroacetic acid) precipitated, and the pellets were resuspended in 2X Laemmli sample buffer. Proteins were separated by sodium dodecyl-sulphate polyacrylamide gel electrophoresis (SDS-PAGE) on NuPage 4–12% Bis-Tris gradient gels and stained using SYPRO Ruby stain according to the manufacturer’s protocol (Thermo Fisher). Proteins from the same C-capsid preparations were again separated by SDS-PAGE and transferred to nitrocellulose membranes using the iBlot2 transfer machine. Membranes were processed for immunoblotting as described above. The primary antibodies used were rabbit R2421 αVP23 [53], mouse monoclonal LP1 for VP16 [58], rabbit 780 αUL37C [59], rabbit 74 αpUL16 [32] and rabbit 121 αpUL21 [31].

Quantitation of protein bands was performed using the iBright 1500 (Invitrogen). Bands were manually drawn, and the values for Local Background Corrected Volume were calculated by the iBright software. For each set of C-capsids, these values from the pUL37 and the VP16 bands were normalized to the Local Background Corrected Volume value of VP23 from the same sample. The normalized values for each virus protein were then compared and analysed using GraphPad Prism 9 software.

## Results

### Cre excision of the BAC-YCp sequence in the mutant viruses

**Fig. 1.**
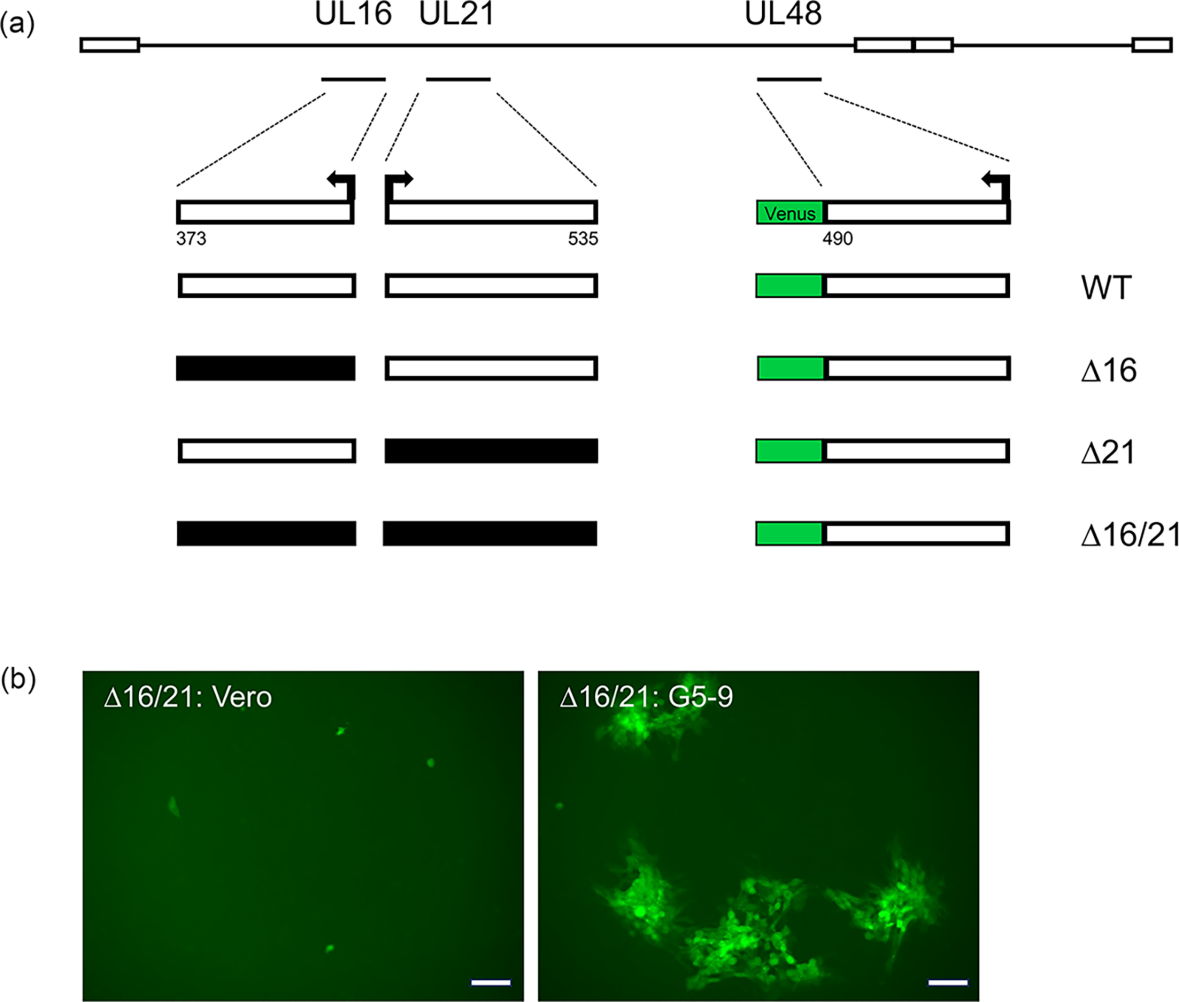
Illustration of the genotypes and phenotypes of the mutant viruses. (**a**) The genomes of the four viruses, WT, ∆16, ∆21 and ∆16/21 (∆:deletion), are shown. The intact ORF (white shading) and the number of codons encoded by each ORF are shown as well as the direction of transcription (arrow). The deletions in the genes (shaded black) for UL16 and UL21 encompassed all the CDSs. The Venus ORF was fused to the C-terminus of VP16. (**b**) Fluorescence image of the replication and spread of ∆16/21 on Vero and G5-9 cell lines (objective 10X). Scale bar=100 µm.

Previously, we had observed that the HSV-1 strain KOS yeast-assembled genome had problems with replication in Vero cells. This was judged to be due to the presence of the BAC-YCp sequence in the virus genome. Removal of the sequence resulted in WT kinetics of virus replication [25]. Because we have the vector sequence bracketed by *loxP* sites, we performed Cre excision on all of our assembled genomes in order to remove the BAC-YCp element. We used an *in vitro* Cre excision method, which gave us an efficiency of ~70% and, more recently, almost 90% [55]. Single plaques were isolated following transfection of cells and screened using PCR assays for excision of the vector sequence. These plaques were used to amplify the virus to obtain a secondary stock and subsequently high-titre working stocks. All these viruses encode a VP16-Venus fusion protein, which enables one to visually follow virus replication (Fig. 1a). This fusion does not affect the ability of the virus to replicate [25]. We typically passage all the mutant viruses in G5-9 cells because of the complementing activity provided in trans. G5 cells were transformed with the EcoR1 G fragment (HSV-1 KOS nucleotides 29281 : 45511) and pSV2neo [53]. This fragment encodes genes UL16–UL21. This cell line can complement mutants in UL16, UL17, UL18, UL19, UL20 and UL21. G5-9 is a subclone of G5 that displays better complementing activity. When the mutant viruses were plaqued on Vero cells, the ∆16 and ∆21 mutant viruses gave rise to small plaques on Vero monolayers. Plaques were not observed on Vero cells when the double mutant virus was plated; only single fluorescent foci were observed (Fig. 1b).

**Fig. 2.**
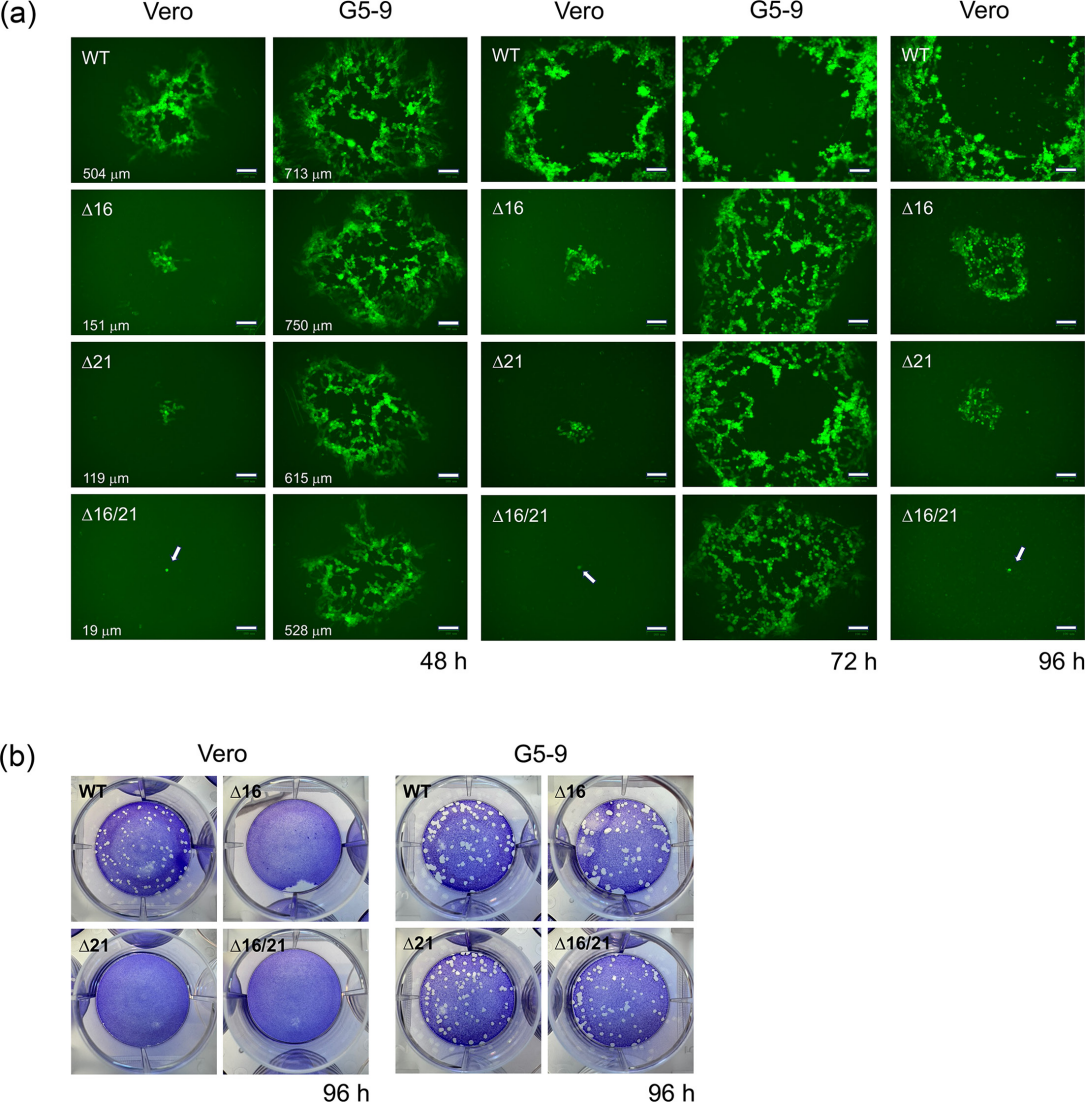
Plaque formation of the mutant viruses on Vero and G5-9 cells. (a) Cell monolayers were infected with 100 p.f.u., and the developing plaques were imaged using fluorescence microscopy. Plaques for the mutants on Vero cells became evident at 48 h. The average diameter in micrometre (*n*=5) of the plaques was measured for plaques that had formed at 48 h. Plaques became too large on G5-9 cells at 96 h to be accurately imaged. Arrows indicate single foci of the ∆16/21 mutant virus on Vero cells. Scale bar=100 µm. (b) The plaques that formed after 96 h post-infection were visualized after crystal violet staining.

We also analysed plaque formation and plaque size of all the mutants in greater detail during incubation over a 96 h period. Vero or G5-9 monolayers were infected with ~100 p.f.u., and the plaques that formed under methylcellulose were imaged over time (Fig. 2a). The plaques that formed on G5-9 cells at 96 h were so large that they could not be fully imaged on the fluorescence Zoe Imager. In the 48 h panel, the average diameter of the plaque (*n*=5) in micrometre for the different mutants is shown. The ∆16 and ∆21 mutant viruses formed very small plaques on Vero cell monolayers. The ∆16/21 mutant was detected as a single fluorescent focus on Vero cells. On G5-9 cell monolayers, the plaques for all the mutants were almost five to ten times larger. Plaques that formed after 96 h are shown in Fig. 2(b) after staining with crystal violet.

### Protein expression

**Fig. 3.**
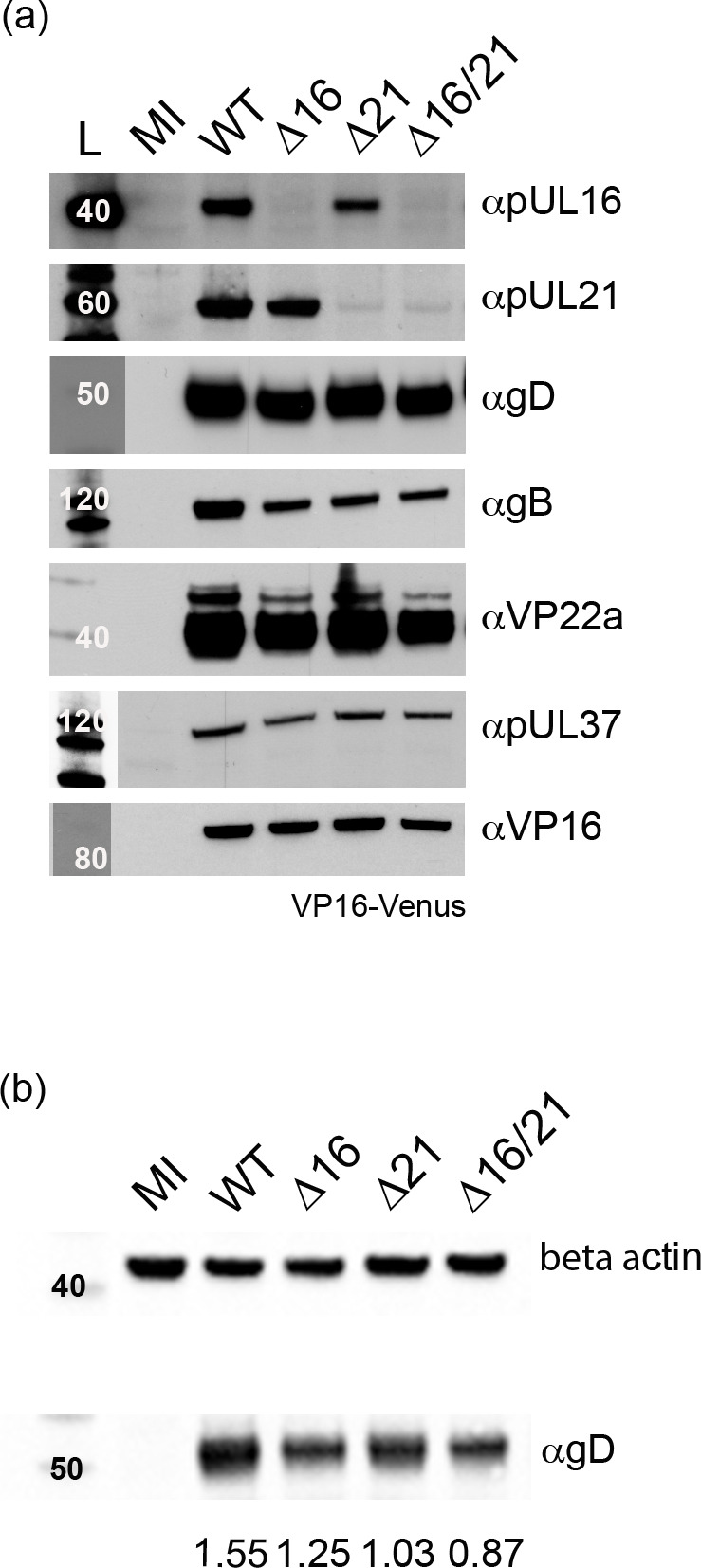
Expression of HSV-1 polypeptides in infected cells. (a) Vero cells were synchronously infected with the indicated viruses or mock-infected (MI). Cell lysates were collected 24 h post-infection and analysed by SDS-PAGE and Western blotting with antibodies for HSV-1 proteins. Both pUL16 and pUL21 are only observed in infected cell lysates for viruses expressing the corresponding WT gene. Additionally, lysates were probed with antibodies for gD, gB, VP22a, pUL37 and VP16, which are all expressed with early and late gene kinetics and are mostly unaltered in mutant virus lysates. Anti-VP16 antibodies detect the VP16-Venus fusion protein (92 kDa). Protein molecular weight standards (kDa) are indicated on the left (L=ladder). The lanes for the ladder for gD, pUL37 and VP16 had to be moved in Photoshop to align them with the other ladder lanes. (b) Vero cells were infected as described above, and the protein lysates after transfer to nitrocellulose were probed with gD and beta-actin antibodies. The signals of the two proteins were quantitated using the iBright 1500 (Invitrogen), and the ratio of gD/beta-actin signal is shown below the blots (mean of two determinations). Molecular weight standards are shown in the leftmost lane.

We used rabbit antiserum against pUL16 and pUL21 to confirm the expression of these proteins or their absence in cells infected with the different mutant viruses. Proteins pUL16 (predicted mass 40 kDa) and pUL21 (predicted mass 58 kDa) were observed in cells infected with the KOS^YA^ WT virus (Fig. 3a). They were not observed in the cell lysates of the corresponding mutant, and both proteins were absent in the double ∆16/21 mutant virus-infected cells. We also examined the expression of other viral proteins in the same lysates. Thus, the levels of gD, gB VP22a, pUL37 and VP16 looked similar in both the WT and single mutant lysates. However, there was a detectable decrease in the levels of protein accumulation in the double mutant. We determined the relative expression of gD to beta-actin using similar immunoblot methods and quantification on an iBright 1500 Imager (Fig. 3b). As the results show, there was a quantifiable decrease in gD levels in ∆16/21 (0.87)-infected cells compared to WT-infected cells (1.55). This was also seen in ∆21-infected cells. This could be due to the effect of pUL21 on gene expression [40,47].

### Growth curves

**Fig. 4.**
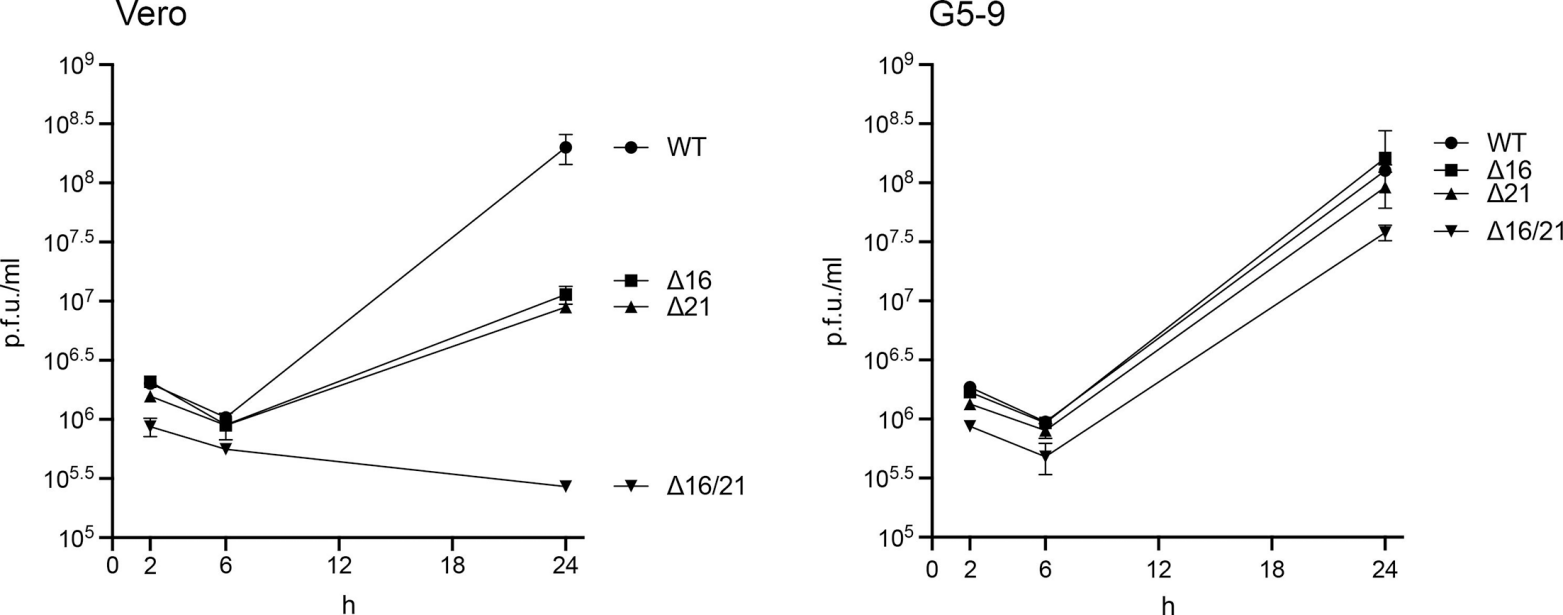
Single-step growth curves of the mutant viruses. Vero and G5-9 cells were infected at an MOI of 10 p.f.u./cell, and the infected cells were harvested at 2, 6 and 24 h post-infection. Virus litres were enumerated by plaquing on G5-9 cells. Data presented are representative of two biological replicates.

In order to quantitate the growth defect in the different mutants, we performed single-step growth assays at high MOI in Vero and G5-9 cells. Both the ∆16 and ∆21 mutant viruses could replicate in Vero cells, but the yields of virus were much lower than in WT virus-infected cells (Fig. 4). For the double mutant, there was no observable virus growth in Vero cells. Growth for all the mutants was recovered in the complementing G5-9 cells and was comparable to WT virus growth except for the ∆16/21 mutant virus, which was slightly lower.

### Cell-to-cell spread of the mutant viruses

**Fig. 5.**
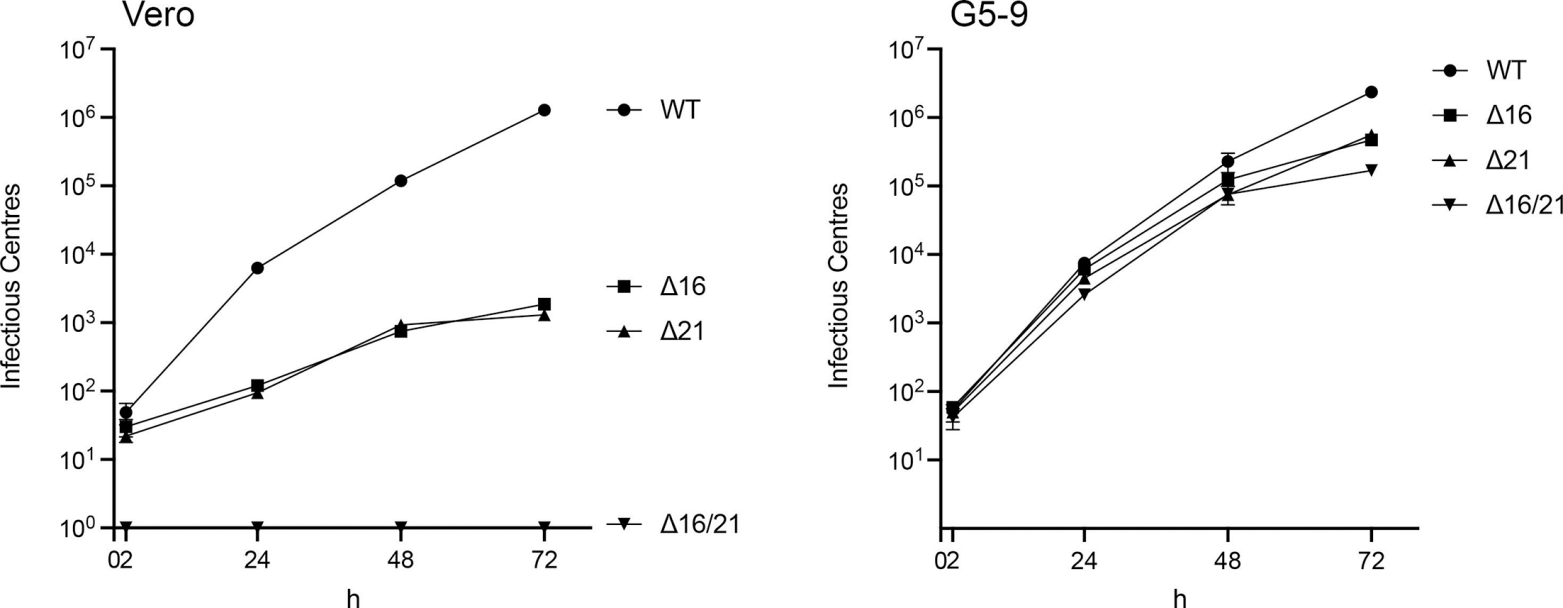
Viruses encoding deletions in UL16 and UL21 are attenuated for cell-to-cell spread in Vero cells. Vero and G5-9 cells were infected with each virus with 100 p.f.u., and the infected cells were trypsinized and harvested at 2 h post-infection and then every 24 h over a 72 h period. The infected cells were mixed with uninfected G5-9 cells and plated to enumerate infectious centres. Data from replicates were plotted.

In order to examine the cell-to-cell spread of the mutant viruses, we performed an infectious centre plaque assay. In this assay, infected cells are harvested at different times post-infection and mixed with uninfected G5-9 cells to enumerate the number of infectious centres. This assay thus accurately measures how the virus spreads from an infected focus into neighbouring uninfected cells. The data observed in Vero cells showed a robust increase in infectious centres for the WT virus and a slower spread for ∆16 and ∆21 mutant viruses. The ∆16/21 mutant was completely unable to spread to neighbouring cells (Fig. 5). In G5-9 cells, all the mutants displayed a robust ability to spread to neighbouring cells.

### Confocal imaging of infected cells

**Fig. 6.**
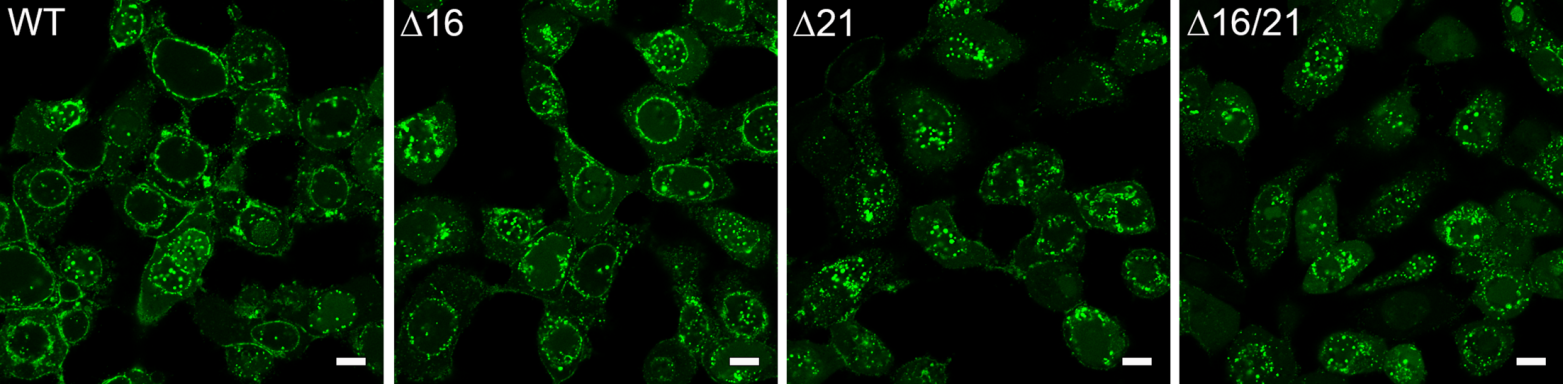
Confocal microscopy reveals disrupted perinuclear VP16-Venus localization in infected cells lacking pUL21. RPE-1 cells were plated in chamber slides and synchronously infected with each virus for 12 h prior to live cell imaging by confocal fluorescence microscopy to visualize VP16-Venus (objective 63X). Perinuclear VP16-Venus was observed in WT virus and Δ16-infected cells, but this distribution became irregular in either the Δ21- or Δ16/21-infected cells. Scale bar=10 µm.

**Fig. 7.**
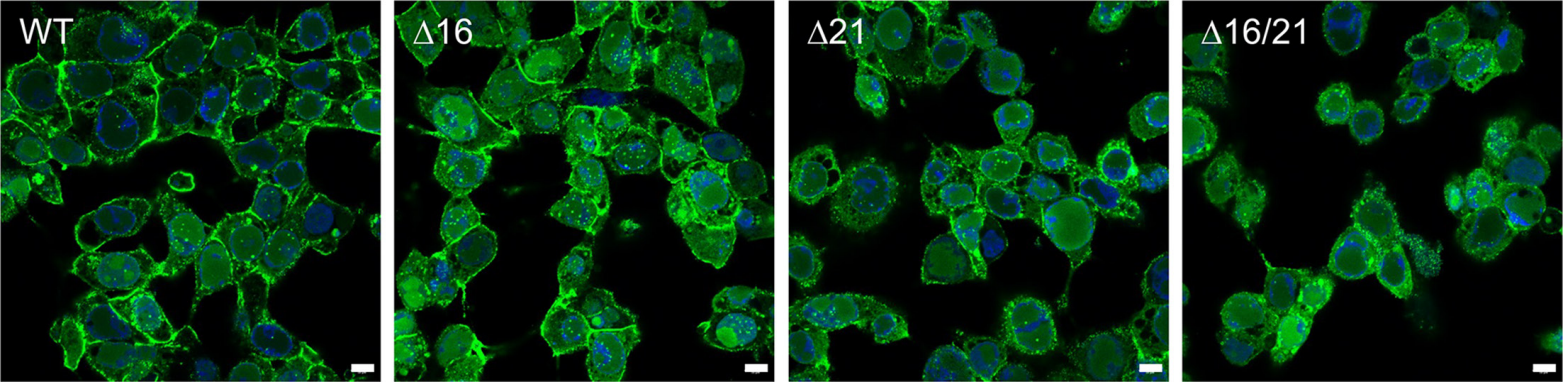
Light microscopy imaging of infected Vero cells. Vero cells in chamber slides were infected with the WT and the mutant viruses at an MOI of 5 p.f.u./cell. The cells were imaged by confocal microscopy at 20 h post-infection after fixation with paraformaldehyde (63X). The overlay of VP16-Venus and the DNA nucleo-blue stain are shown. Scale bar=10 µm.

Because the mutant viruses have the VP16-Venus tag in their genomes, we could visualize the fluorescence distribution using confocal light microscopy. For this, RPE-1 cells were used and infected at a high MOI. Cells were imaged at 12 h post-infection. VP16-Venus has a nuclear punctate distribution early in the infection, but as time progresses, fluorescence was visualized at the nuclear and cytoplasmic membranes, including the plasma membrane. In the cells infected with the ∆16 mutant virus, the distribution of fluorescence was similar to WT. In the cells infected with ∆21 and ∆16/21 mutant viruses, the distribution of VP16 was perturbed and was less localized to the nuclear and cytoplasmic membranes. This was more evident for the double mutant virus (Fig. 6). We also performed the same experiment in Vero cell monolayers (Fig. 7). For both the WT virus and ∆16, we observed cell surface fluorescence, indicative of mature virus. For ∆ 21, there was diminished cell surface fluorescence and absent in ∆16/21-infected cells. Similar to RPE-1 cells, the nuclear membrane fluorescence observed in ∆21 and more so in ∆16/21-infected cells was perturbed.

**Fig. 8.**
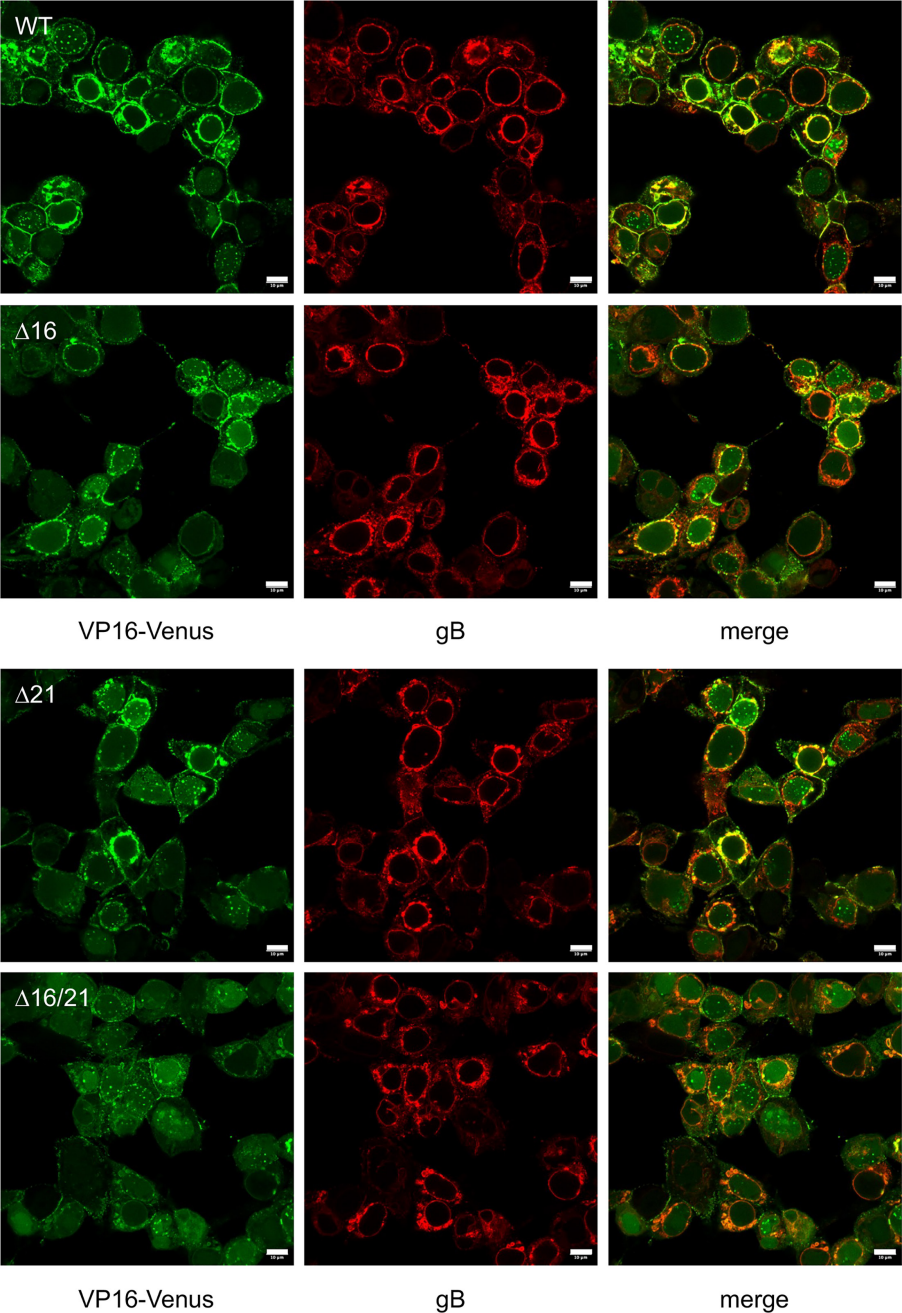
Immunofluorescence imaging of VP16-Venus and gB in Vero-infected cells. Vero cells were infected as in the legend to Fig. 7. At 20 h post-infection, the cells were fixed, permeabilized and stained for gB (red) and imaged using confocal microscopy. Scale bar=10 µm.

We carried out immunofluorescence experiments using antibodies to gB. The Vero cells were permeabilized for this assay, and data derived for gB are shown (Fig. 8). What was noticeable was the nuclear membrane localization of gB. In both WT and ∆16-infected cells, the nuclear membrane fluorescence was distinct and, in some cells, merged with the VP16-Venus fluorescence. This was less pronounced in ∆21 and ∆16/21-infected cells. What was evident was the fluorescence of gB in what appears to be structures as a result of nuclear membrane blebbing or disruption. This was evident in some WT-infected cells but was greatly increased in ∆16/21-infected cells.

### Ultrastructural analysis of infected cells

**Fig. 9.**
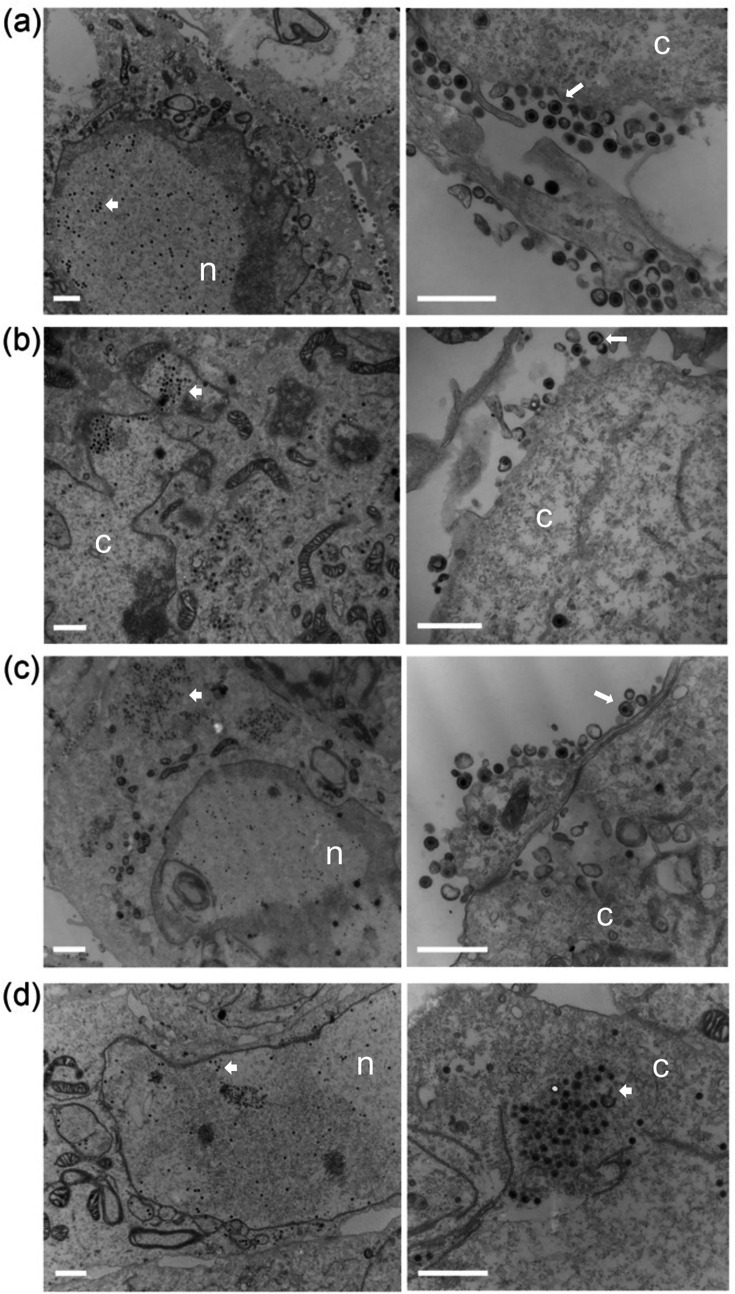
TEM shows enveloped capsids in single Δ16 or Δ21 null virus-infected cells, but not in Δ16/21-infected cells. Vero cells were synchronously infected with WT (a), Δ16 (b), Δ21 (c) or Δ16/21 (d) viruses, then fixed 16 h post-infection and processed for TEM imaging. Enveloped virus particles were observed in the cytoplasm and egressing from WT-infected cells (white arrows) and observed at a lower frequency in Δ16- or Δ21-infected cells. Enveloped virus particles were not observed in Δ16/21 cells, and an accumulation of unenveloped capsids (white arrowheads) was observed in the cytoplasm of these cells. The nucleus (n) and cytoplasm (c) are marked. Scale bar=1 um.

**Fig. 10.**
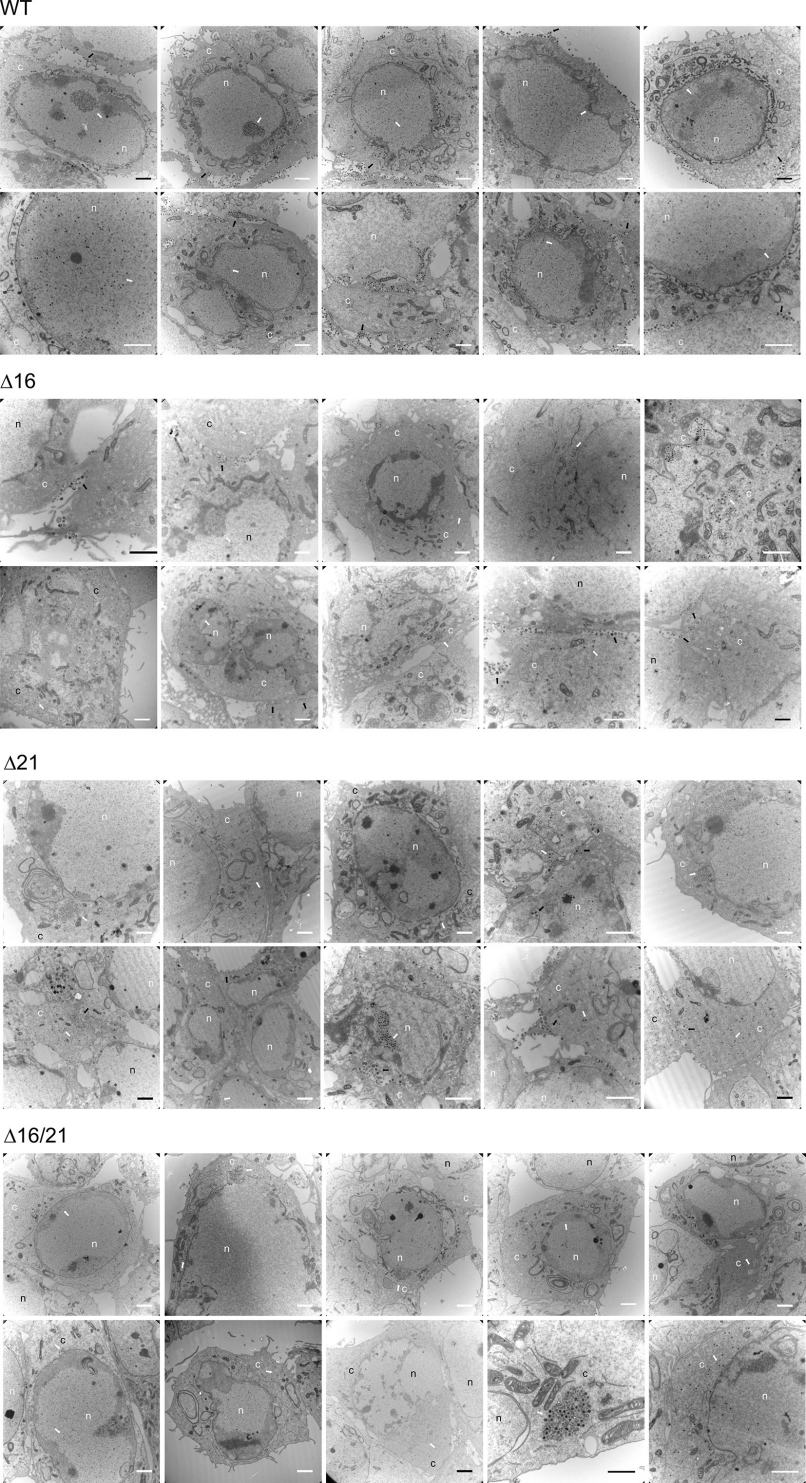
Transmission electron microscopy of infected cells. Images presented were taken at lower magnification to show the distribution and numbers of capsids and enveloped particles. The nucleus (n) and cytoplasm (c) compartments are marked. Capsids are indicated by a white arrow and enveloped particles by a black arrow. Scale bar = 2 um.

Vero cells were infected with all the mutant viruses and examined by electron microscopy to visualize in greater detail what was happening within the cell (Fig. 9). For the WT-infected cells, microscopy showed capsids in the nucleus, enveloped virus in the cytoplasm and at the cell surface, which is typical of productive virus production (Fig. 9a). In the cells infected with ∆16 (Fig. 9b) and ∆21 (Fig. 9c) mutant viruses, capsids were evident in the nucleus as well as in the cytoplasm. There were fewer enveloped viruses observed, which reflects the lower production of virus in these cells. For the double mutant-infected cells, we observed capsids in the nucleus as well as in the cytoplasm (Fig. 9d). However, there were no enveloped viruses detected in these cells. Also shown in Fig. 10 are additional TEM images at lower magnification of infected cells (ten panels for each virus). These can be used to judge the distribution and state of capsid structures: nuclear versus cytoplasmic versus enveloped particles at the cell surface. What was evident from these images is that in WT-infected cells, there are large numbers of enveloped particles at the cell surface plasma membrane or between adjacent cells. There were fewer such enveloped particles in the ∆16- and ∆21-infected cells at the plasma membrane; however, no enveloped particles were evident in ∆16/21-infected cells. In the ∆16/21 mutant-infected cells, non-enveloped cytoplasmic capsids were evident, indicating defects in maturation in this compartment. For all infected cells, nuclear capsids were evident, but we did not see these capsids accumulating in this compartment, which is indicative of a potential nuclear egress defect. There were some cells (∆16/21) in which only nuclear capsids were evident, but these were distributed throughout the nucleus area.

### Capsid assembly

**Fig. 11.**
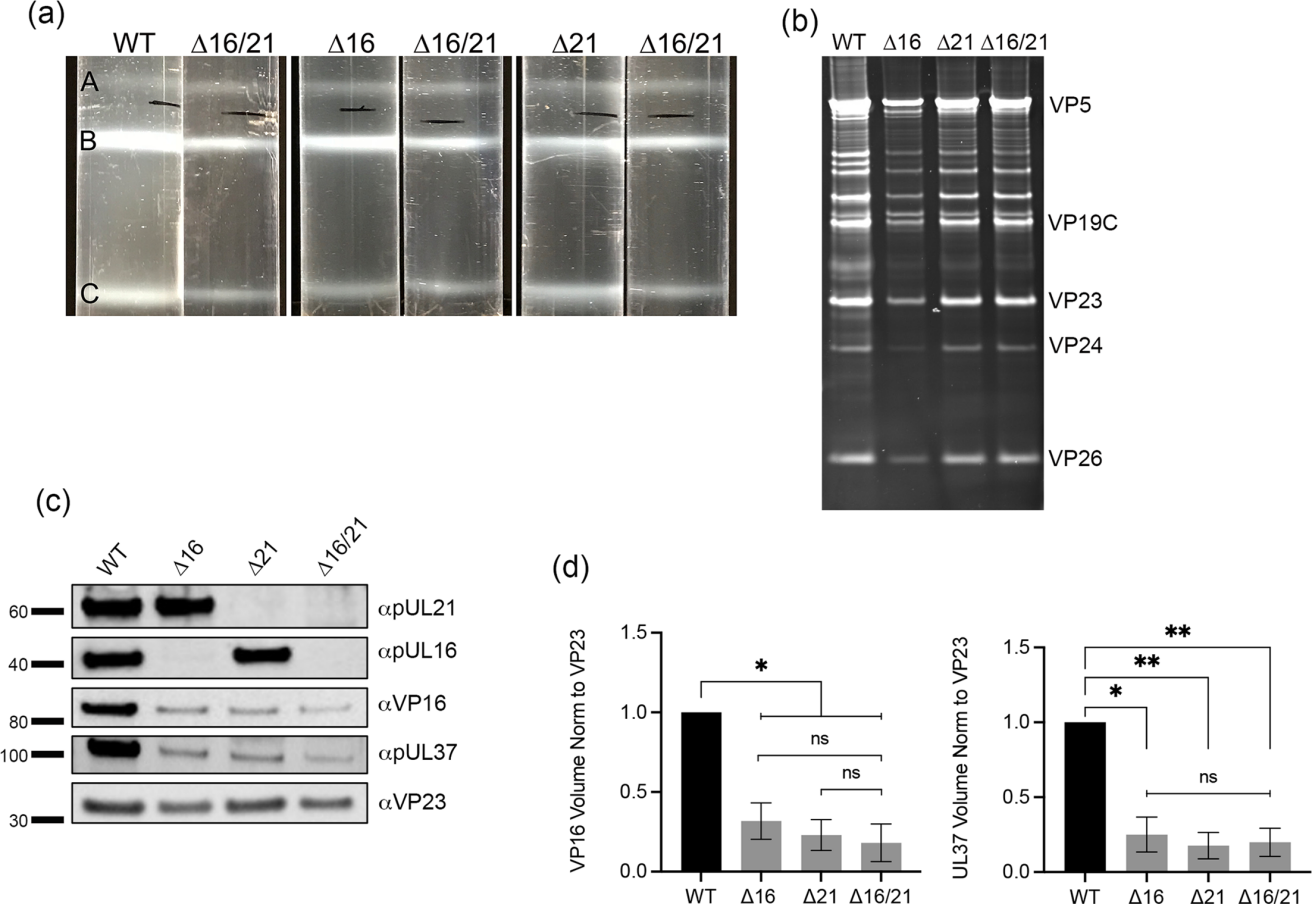
Isolation and analysis of mutant capsid particles. Vero cells were infected with each virus (MOI=5), and infected cell pellets were collected 24 h post-infection. Capsids from infected cells were released by treating infected cell pellets with 2X CLB and sonication followed by separation of capsids on 20–50% sucrose gradients and ultracentrifugation. (a) Each capsid form – A, B and C – is observed as light-scattering bands and denoted on the gradient images, each compared to the Δ16/21 capsid bands. (b) C-capsids were harvested by side puncture from each gradient, and proteins were separated by SDS-PAGE and observed by SYPRO Ruby staining. Visible capsid protein identities are indicated. (c) Proteins from the same C-capsids were again separated by SDS-PAGE and probed for pUL16 or pUL21 by Western blotting as well as the capsid triplex protein VP23. Additionally, capsid proteins were also probed for inner-tegument protein pUL37 or outer-tegument protein VP16 (Venus fusion). Molecular standards (kDa) are indicated on the left. (d) Quantitation of levels of VP16 and pUL37 detected in the C-capsid fractions relative to the triplex protein, VP23. Western blots were analysed using the iBright 1500, yielding values of the Local Background Corrected Volume for each protein band. The VP16 and pUL37 volumes were normalized to the VP23 volumes and then normalized to the WT capsids. Statistical analyses were performed with GraphPad Prism 9 using Student’s t-test. ns: not significant, *P*=<0.05 (*, ** and ***).

We next examined capsid assembly and composition using purified capsids (Fig. 11). Whole cell lysates were sedimented through sucrose gradients, and all three capsid types (A, B and C) were observed (Fig. 11a). There were lower levels of capsids in the gradients using lysates for ∆16/21-infected cells as judged by light scatter. We extracted the C-capsids and analysed these using total protein stain (Fig. 11b). One could readily identify the major capsid proteins; however, the levels of these and thus C-capsids were generally also lower in the double mutant lysate gradients. We have analysed a number of capsid gradients following replicate infections. The experiment shown in Fig. 11(b) shows lower levels of capsids in ∆16 virus cell lysates, which was not commonly seen. We next examined these C-capsids for their composition using available antibodies to the different tegument and capsid proteins (Fig. 11c). We chose to normalize our capsids using an antibody to VP23, which is present in capsids in a fixed amount (600 copies). When antibodies to VP16 and pUL37 were used, we observed a significant decrease in the amounts detected relative to VP23 normalization in all the mutant capsids. This was examined using the quantitation software in the iBright 1500 and analysed using GraphPad Prism 9 software (Fig. 11d). There was a significant reduction in the capsid association of both VP16 and pUL37 in the mutant capsids. This same observation was observed in multiple replicate analyses of C-capsids isolated from infected cells using the same methods.

## Discussion

Proteins pUL16 and pUL21 have been identified in different and varied activities in the infected cell. The UL21 gene product has RNA binding activity, is involved in syncytial processes, affects US3 kinase activity, inhibits innate immunity signalling, acts as a viral phosphatase and alters host metabolic pathways [48,60,65]. Protein UL16 similarly displays a variety of activities in the cell including interactions with host mitochondria [66,67] as well as a role in syncytial formation [68]. Both pUL16 and pUL21 exhibit dynamic interactions with both tegument and glycoproteins of the virus [5,28, 29, 32,35, 43, 68]. These different activities illustrate the complexity of the functions of these two proteins in HSV-infected cells.

Deletion of each gene individually has been done in a number of HSV strains with differing results [40,41, 43, 46, 47, 72,74]. In most cases, deletion of the gene in HSV-1 does not significantly affect virus replication, albeit it can impact the levels of virus production and spread. In HSV-2, the single deletion of UL16 or UL21 affects nuclear egress and retention of the viral genome in the assembled capsid [46,49, 50]. A comprehensive analysis of mutants in these two genes in both HSV-1 and HSV-2 strains has been done by the Banfield lab [73,74]. These results show that there are significant differences between the two strains as well as within strains. UL21 mutants in HSV-1 strains KOS and F and HSV-2 strain SD90e displayed a tenfold reduction in replication/spread. UL21 mutants in HSV-2 strains HG52 and 186 displayed a 100-fold and 200-fold reduction in replication, respectively [73]. The authors note that these types of measurement of virus replication differed in different cell types. These differences were also noted in side-by-side analyses of UL16 mutants [74]. The type 2 strains were more debilitated in virus spread than the HSV-1 strains. This illustrates the complexity of these two gene products and how they function in different genotypes as well as in different cell culture systems. Our findings show that the single mutants display defects in virus production in cells as well as in cell-to-cell spread. One observation of note is that the mutant viruses are more attenuated for cell-to-cell spread than single-step growth. The double mutant cannot produce infectious progeny, and as a consequence, it cannot generate infectious foci.

Initial envelopment of the HSV-1 virion takes place at the inner nuclear membrane (INM). The NEC, which includes the interacting proteins, pUL31 and pUL34, is required for this initial envelopment, as reviewed in [9,10, 75,81] as well as, in some situations, the US3 kinase. After the capsid is enveloped at the INM, it fuses with the outer nuclear membrane depositing a naked (non-enveloped) particle into the cytoplasm [82]. This process is significantly altered by the UL16 and UL21 gene products, because in HSV-2-infected cells, mutants in these two genes have defects in nuclear egress [49]. Studies from the Banfield lab have shown in detail how HSV-2 pUL21 alters the activity of the NEC [48,49]. Absence of pUL21 results in hyperphosphorylation of pUS3 and consequently of the NEC. This results visually in disruptions/perturbations of the architecture of the nuclear membrane. This was also observed when an HSV-1 strain with a UL21 deletion was analysed or a virus carrying a deletion of pUS3 kinase [49]. In this study, we have also observed nuclear membrane perturbations in cells infected with ∆21 and ∆16/21 as judged by VP16-fluorescence and gB nuclear membrane fluorescence. These alterations were seen in WT- and ∆16-infected cells but to a much lesser extent. This structural change could affect how the virus gets out of the nucleus. The lack of functional NEC could slow the rate of nuclear exit as well as change the protein incorporation in the exiting capsid. Nuclear membrane disruptions could result in the sequestration of proteins important for virus maturation away from the budding sites in the nuclear envelope. We do observe capsids that get out into the cytoplasm of infected cells, so we do not believe that there is a significant impediment to capsid translocation out of the nucleus. We did recombine the VP26-mCherry tag [83] into the genomes of these mutant viruses. When the infected cells were examined, the nuclear assembly sites visualized by the red fluorescence were similar to WT, and there was not a detectable change in their localization (data not shown). It was difficult to follow individual exiting capsids because the strong signal from the assembly sites overwhelmed the detection and thus the ability to discern such particles.

In relation to our analyses, the Banfield group made interesting observations on how capsids may be localizing in the mutant-infected cells. Using a similar mCherry tag on VP26 and a VP5 antibody, they observed sequestration of signal that was aberrant from WT signal in the nuclear membrane, and they show that capsids were trapped at the cytoplasmic face of the nuclear membrane colocalized with nuclear pores [51]. This is seen with HSV-2 and HSV-1 single mutant-infected cells as well as in an HSV-1 KOS mutant they generated that had both UL16 and UL21 mutated. Using ectopic expression of pUL16 and pUL21, they could block capsid association. These findings raise interesting questions about the activities of these proteins at the nuclear membrane both during exit and entry.

The protein composition of C-capsids analysed in our investigation revealed interesting findings. All the mutant C-capsids had reduced levels of pUL37 and VP16 compared to WT capsids. The double mutant has the lowest levels of incorporation. Both pUL37 and VP16 play essential roles in virus maturation, which could explain the defects in virus maturation. A more comprehensive analysis of the protein composition of the mutant capsids is required to begin to formulate mechanistically how these particles mature. Since all three mutant capsids had reduced levels of pUL37 and VP16, the nuclear membrane perturbations cannot completely explain this finding, since in ∆16-infected cells (Fig. 6) and as reported by Gao *et al*., the nuclear membrane has a more normal architecture [49]. Capsid association for the interacting proteins, pUL16 and pUL21, was different. The levels of pUL21 incorporated into ∆16 mutant capsids were similar to those detected in WT capsids, and conversely, ∆21 capsids had similar levels of pUL16 compared to WT capsids. The other documented experiments that looked at the protein composition of capsid particles relevant to this discussion were done by the Banfield lab who showed that the levels of pUL16 were lower in C-capsids isolated from HSV-1 KOS ∆UL21 and HSV-2 ∆UL21-infected cells [50]. This difference could be due to differences in isolation methods of capsids. Of note, the capsid gradient image that they present shows a broad light-scattering band for C-capsids [50]. We generally observe a more tight band for C-capsids (Fig. 11).

Most observations demonstrate that the tegument primarily matures in the cytoplasm, and sequential interactions between capsid-tegument, tegument-tegument and tegument-envelope drive the assembly of this structure [29,84]. One of the key questions is what complex initiates tegument assembly. Data have been interpreted that suggest [85,87] that it is the largest tegument protein, pUL36, that initiates this. Data have also demonstrated the role of VP16 in complex with VP22, pUL41 and pUL47 as the ‘organisers’ of the tegument [29,86, 88,90]. Data have also implicated that the complex comprised of pUL11, pUL16 and pUL21 playing a pivotal role in linking capsids with the envelope [28,30,32, 35, 36, 91].

In our study, it is evident that pUL21 as well as pUL16 mediate important interactions that affect capsid association of tegument proteins. This includes the inner-tegument protein pUL37 and a major constituent of the tegument, VP16. The reduction of tegument protein incorporation affects robust virus replication (as in the case of the single deletions) or completely abolishes virus replication and spread (as in the case of the double deletions). Both pUL16 and pUL21 proteins have extensive interactions with tegument and envelope proteins. pUL16 has been shown to interact with VP16 as well as gE, pUL11 and VP22 [28,32,35, 43] and is required for the virion incorporation of gD [70]. Thus, the pUL16–pUL21 complexes could be required for bridging interactions between the inner and outer teguments and subsequently with the envelope during secondary envelopment.

## Abbreviations

∆: deletion
CLB: capsid lysis buffer
gB: glycoprotein B
gD: glycoprotein D
HSV-1: herpes simplex virus type-1
INM: inner nuclear membrane
MOI: multiplicity of infection
NEC: nuclear egress complex
ORF: open reading frame
PFU: plaque forming unit
RPE-1: retinal pigment epithelial
SDS-PAGE: sodium dodecyl-sulphate polyacrylamide gel electrophoresis
TAR: transformation associated recombination
TCA: trichloroacetic acid
TEM: transmission electron microscopy
